# Dysfunctional epigenetic protein-coding gene-related signature is associated with the prognosis of pancreatic cancer based on histone modification and transcriptome analysis

**DOI:** 10.1038/s41598-022-27316-2

**Published:** 2023-01-04

**Authors:** Xiao Yu, Yun Wang, Xiaoyi Shi, Zhihui Wang, Peihao Wen, Yuting He, Wenzhi Guo

**Affiliations:** 1 Department of Hepatobiliary and Pancreatic Surgery, The First Affiliated Hospital of Zhengzhou University, No. 1 Jianshedong Road, Erqi District, Zhengzhou, 450052 China; 2 Key Laboratory of Hepatobiliary and Pancreatic Surgery and Digestive Organ Transplantation of Henan Province, The First Affiliated Hospital of Zhengzhou University, Zhengzhou, 450052 China; 3 Open and Key Laboratory of Hepatobiliary & Pancreatic Surgery and Digestive Organ Transplantation at Henan Universities, Zhengzhou, 450052 China; 4 Henan Key Laboratory of Digestive Organ Transplantation, Zhengzhou, 450052 China

**Keywords:** Cancer epidemiology, Gastroenterology

## Abstract

Emerging evidence suggests that epigenetic alterations are responsible for the oncogenesis and progression of cancer. However, the role of epigenetic reprogramming in pancreatic cancer is still not clear. In this study, we used the limma R package to identify differentially expressed protein-coding genes (PCGs) between pancreatic cancer tissues and normal control tissues. The cell-type identification by the estimating relative subsets of RNA transcripts (CIBERSORT) package was used to quantify relative cell fractions in tumors. Prognostic molecular clusters were constructed using ConsensusClusterPlus analysis. Furthermore, the least absolute shrinkage and selection operator and stepAIC methods were used to construct a risk model. We identified 2351 differentially expressed PCGs between pancreatic cancer and normal control tissues in The cancer genome atlas dataset. Combined with histone modification data, we identified 363 epigenetic PCGs (epi-PCGs) and 19,010 non-epi-PCGs. Based on the epi-PCGs, we constructed three molecular clusters characterized by different expression levels of chemokines and immune checkpoint genes and distinct abundances of various immune cells. Furthermore, we generated a 9-gene model based on dysfunctional epi-PCGs. Additionally, we found that patients with high risk scores showed poorer prognoses than patients with low risk scores (*p* < 0.0001). Further analysis showed that the risk score was significantly related to survival and was an independent risk factor for pancreatic cancer patients. In conclusion, we constructed a 9-gene prognostic risk model based on epi-PCGs that might serve as an effective classifier to predict overall survival and the response to immunotherapy in pancreatic cancer patients.

## Introduction

Pancreatic cancer remains one of the deadliest malignancies, with a 5-year survival rate of only 11%^1–3^. It causes 496,000 new cases and 466,000 deaths every year, ranking as the seventh leading cause of cancer-related death worldwide in 2020^4^. Ninety percent of pancreatic cancer patients have no clinical manifestation, and a lack of effective early diagnostic methods leads to this disease ranking first among asymptomatic patients^5^. Pancreatic cancer can be divided into a variety of pathological types. Pancreatic ductal adenocarcinoma is the most common tissue type with the worst prognosis, accounting for 85–90%^6^. Although diagnostic approaches, perioperative management, and systemic therapies have improved, the median survival time of patients is still less than 1 year^1^. Therefore, it is necessary to develop new diagnostic and prognostic biomarkers.

In recent years, many studies have focused on immunotherapy, such as immune checkpoint inhibition, for advanced malignancies. However, pancreatic cancer is less responsive to immune checkpoint inhibitors, and it is a major challenge to identify which patients would be more likely to benefit from immunotherapy. One of the important factors influencing the response to immunotherapy is the tumor microenvironment (TME)^7–10^. The TME is complex and maintained by dynamic cell–cell interactions. Recent studies based on public databases have uncovered marked effects of tumor-infiltrating levels of various types of immune cells on patient prognosis and immunotherapeutic response^11–13^. A better understanding of the characteristics of the TME and how tumors escape the immune system might provide novel clues for the diagnosis and treatment of pancreatic cancer.

Genetic and epigenetic alterations are two major mechanisms that lead to tumorigenesis. Emerging evidence suggests that epigenetic changes are responsible for the progression of cancer^14–16^. Epigenetic modification refers to not changing the sequence of DNA but changing the expression and function of genes and is mainly divided into DNA methylation, histone modification, and chromosome remodeling^17,18^. Among these modifications, histone modification is the most well-known epigenetic modification. However, the role of epigenetic reprogramming in pancreatic cancer is still not clear. A better understanding of the aberrations in epigenetic modification might provide an early diagnostic and prognostic biomarker of pancreatic cancer.

Here, we found epigenetically abnormal genes in pancreatic cancer based on a public database by comparing histone modifications on protein-coding gene (PCG) promoter and enhancer elements, including H3K27ac, H3K27me3, H3K36me3, H3K4me1, H3K4me3 and H3K9me3. We identified 363 pancreatic cancer-specific epigenetically dysregulated genes. Finally, based on these epigenetic PCGs (epi-PCGs), we performed molecular typing of pancreatic cancer samples and identified epi-PCGs that were prognostic markers for pancreatic cancer. Our research results contribute to a better understanding of the role of epi-PCG dysregulation in pancreatic cancer.

## Results

### The genomic landscape of epigenetically dysregulated PCGs

**Figure 1:**
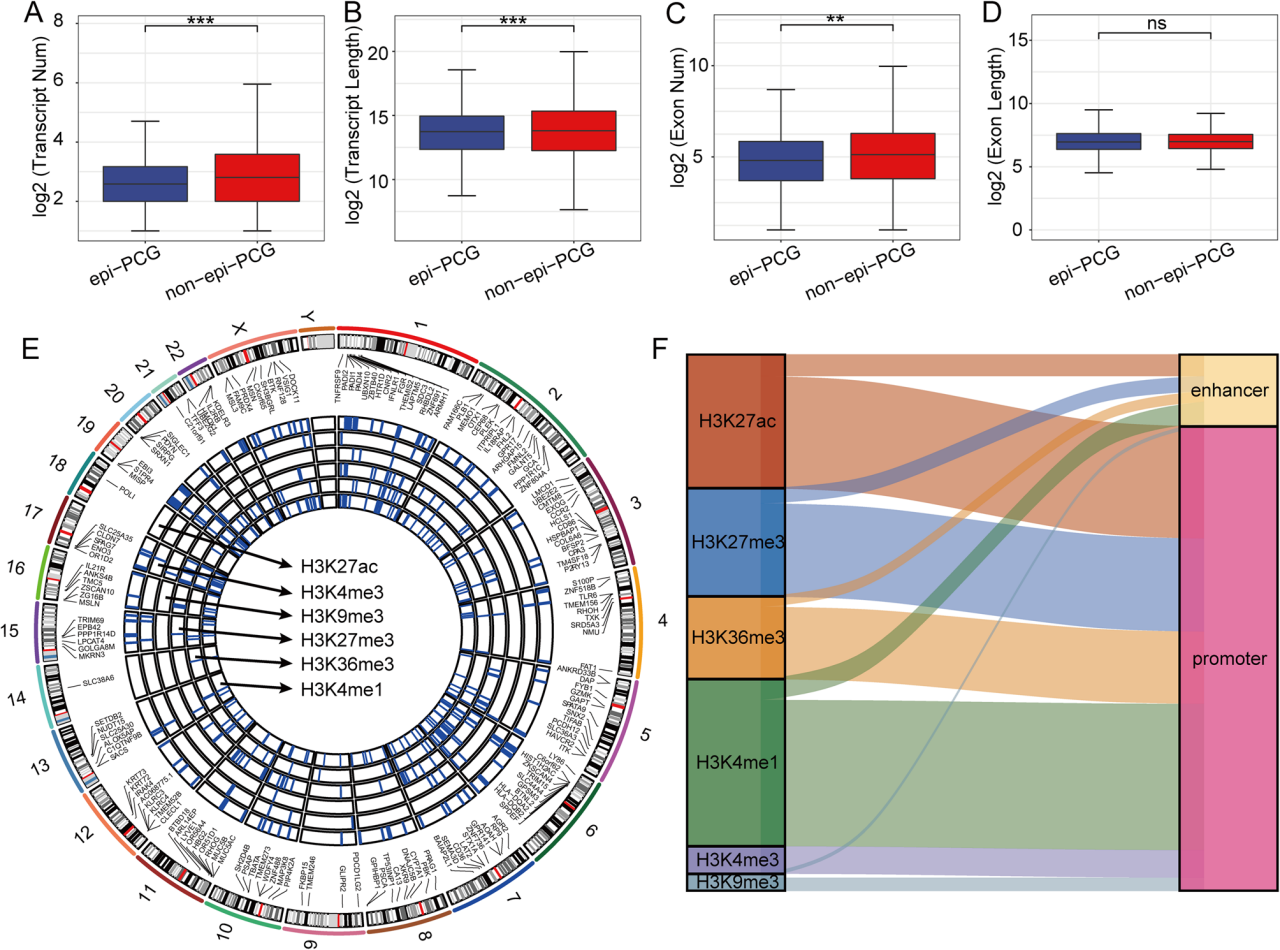
Genomic landscape of epigenetically dysregulated protein-coding genes (PCGs). (**A**) The number of epi-PCG transcripts was significantly lower than that of non-epi-PCG transcripts. (**B**) The length of epi-PCG transcripts was smaller than that of non-epi-PCG transcripts. (**C**) Epi-PCGs had a smaller number of exons than non-epi-PCGs. (**D**) The exons were not different in length between epi-PCGs and non-epi-PCGs. (**E**) Genomic landscape of epi-PCGs induced by different histone modifications. (**F**) Type distribution of dysfunctional epi-PCGs. (*P < 0.05; **P < 0.01; ***P < 0.001).

First, we identified 2351 differentially expressed PCGs between pancreatic cancer tissues and normal control tissues in TCGA. Combined with histone modification data, we ultimately identified 363 epi-PCGs and 19,010 non-epi-PCGs. Epi-PCGs accounted for only 1.87% of all PCGs (363/19,373). We compared the number and length of gene exons and transcripts of epi-PCGs and non-epi-PCGs to characterize the genomic characteristics of PCGs with epigenetic dysfunction. The results showed that the number of epi-PCG transcripts was significantly lower than that of non-epi-PCG transcripts (Fig. 1A), and the length of epi-PCG transcripts was smaller than that of non-epi-PCG transcripts (Fig. 1B). In addition, epi-PCGs had a smaller number of exons than non-epi-PCGs (Fig. 1C), while the exons were not different in length between epi-PCGs and non-epi-PCGs (Fig. 1D). Next, we found that the abnormal histone modifications in these PCGs were mainly H3K27ac, H3K27me3, H3K36me3, H3K4me1, H3K4me3, and H3K9me3 (Fig. 1E), and most of the apparently dysregulated PCGs were accompanied by a variety of histone modification abnormalities (Fig. 1E). Notably, these abnormal histone modifications were mainly concentrated in the promoter region (Fig. 1F).

### Functional enrichment analysis of dysregulated epi-PCGs

To characterize the functions of dysregulated PCGs, we systematically analyzed the correlation between the epi-PCGs and the pathways in pancreatic cancer. We calculated the enrichment scores of these PCGs for each sample using ssGSEA. The results showed that the GSEA scores of dysregulated histone scores in H3K9me3 enhancers, H3K9me3 promoters and H3K27me3 enhancers in tumor samples were significantly lower than those in healthy samples (Fig. S1A), suggesting that dysregulated histone genes have tumor suppressor functions in cancer.

**Figure 2:**
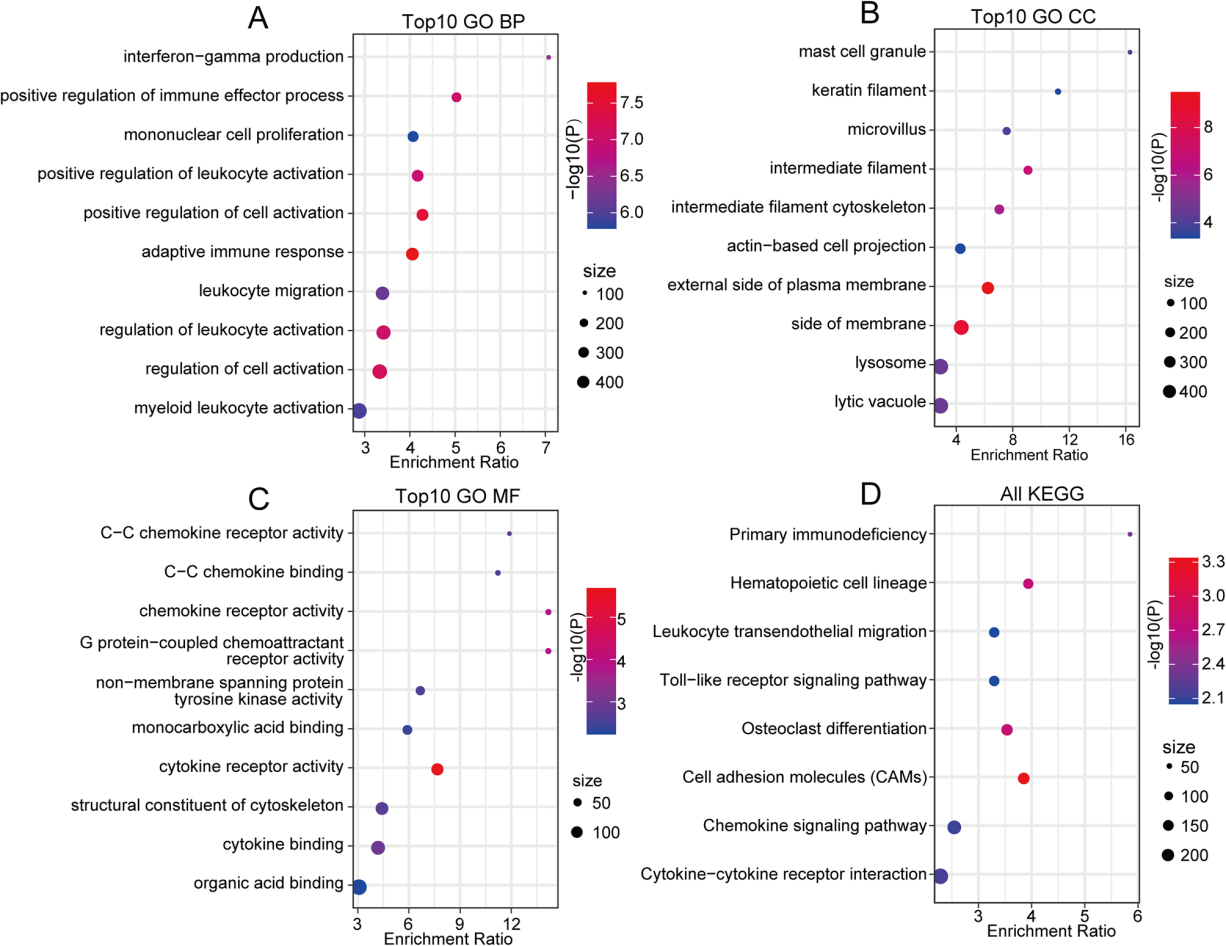
KEGG and GO analyses of dysregulated epi-PCGs. (**A**) BP annotation map of epi-PCGs. (**B**) MF annotation map of epi-PCGs. (**C**) CC annotation map of epi-PCGs. (**D**) KEGG annotation map of epi-PCGs. (BP: biological process; MF: molecular function; CC: Cell component).

Furthermore, we evaluated the relationship between the enrichment score of each type of epi-PCG and KEGG pathways to obtain the relevant KEGG pathways of each type of epi-PCG. The most relevant KEGG pathways of the 11 types of epi-PCGs are shown in Fig. S1B^19^. Among these 41 pathways, there were tumor-related pathways, including the p53 signaling pathway, ECM–receptor interaction, pancreatic cancer, pathways in cancer, and immunization-related pathways. All these results indicated that epi-PCGs were closely related to the occurrence, development and metabolism of pancreatic cancer. Next, KEGG pathway analysis and GO functional enrichment analysis were performed for epi-PCGs. The top 10 annotations with significant differences in the biological process (BP), cellular component (CC) and molecular function (MF) categories are shown in Fig. 2A–C. For epi-PCG KEGG pathway enrichment, a total of 8 pathways were significantly annotated (*p* < 0.05, Fig. 2D).

### The relationship between dysregulated epi-PCGs and RNA modification

RNA modifications have recently been considered essential regulators of coding and noncoding RNA processing and function and affect various biological processes^20^. Among RNA modifications, 6-methyladenosine (m6A) is the most abundant and better characterized modification in RNA, and m5C and m1A are prevalent markers of RNA^21–23^. Here, we extracted m6A, m5C, and m1A gene expression data from the TCGA-PACA cohort. We found a close correlation between the enrichment scores of 11 types of epi-PCGs and the m6A, m5C, and m1A genes (Fig. S2).

### Construction of prognostic molecular clusters based on epi-PCGs

**Figure 3:**
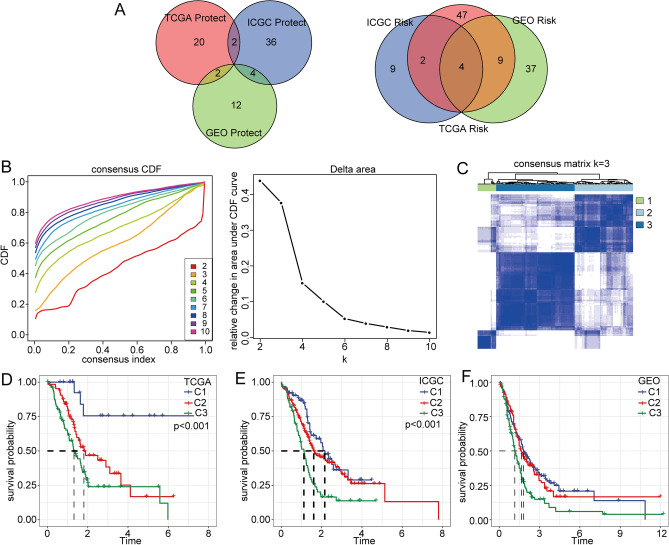
Construction of prognostic molecular clusters based on epi-PCGs. (**A**) Venn diagram of prognostic-related genes screened by univariate Cox regression analysis. (**B**) CDF curve of samples from the TCGA-PACA cohort and CDF delta area curve, delta area curve of consensus clustering. (**C**) Sample clustering heatmap when consensus k = 3. Survival analyses for the three clusters based on patients with pancreatic cancer from TCGA dataset (**D**), ICGC dataset (**E**), and GEO dataset (**F**).

First, we performed univariate analysis of the epi-PCGs of pancreatic cancer in the TCGA, ICGC and GEO datasets and screened prognostic genes (*p* < 0.05). The criteria for screening prognostic genes were survival related in at least two data sets. A total of 23 genes were identified. (Fig. 3A). Then, the 23 genes were clustered by ConsensusClusterPlus analysis, and the optimal number of clusters was determined by the CDF. Finally, we chose k = 3 and obtained three epi-PCG-related clusters (Fig. 3B–C). Moreover, Kaplan–Meier analysis showed that different clusters had different overall survival (OS) rates in pancreatic cancer based on the TCGA, ICGC, and GEO databases (Fig. 3D–F).

### Expression of chemokines and immune checkpoint genes in different epi-PCG clusters

**Figure 4:**
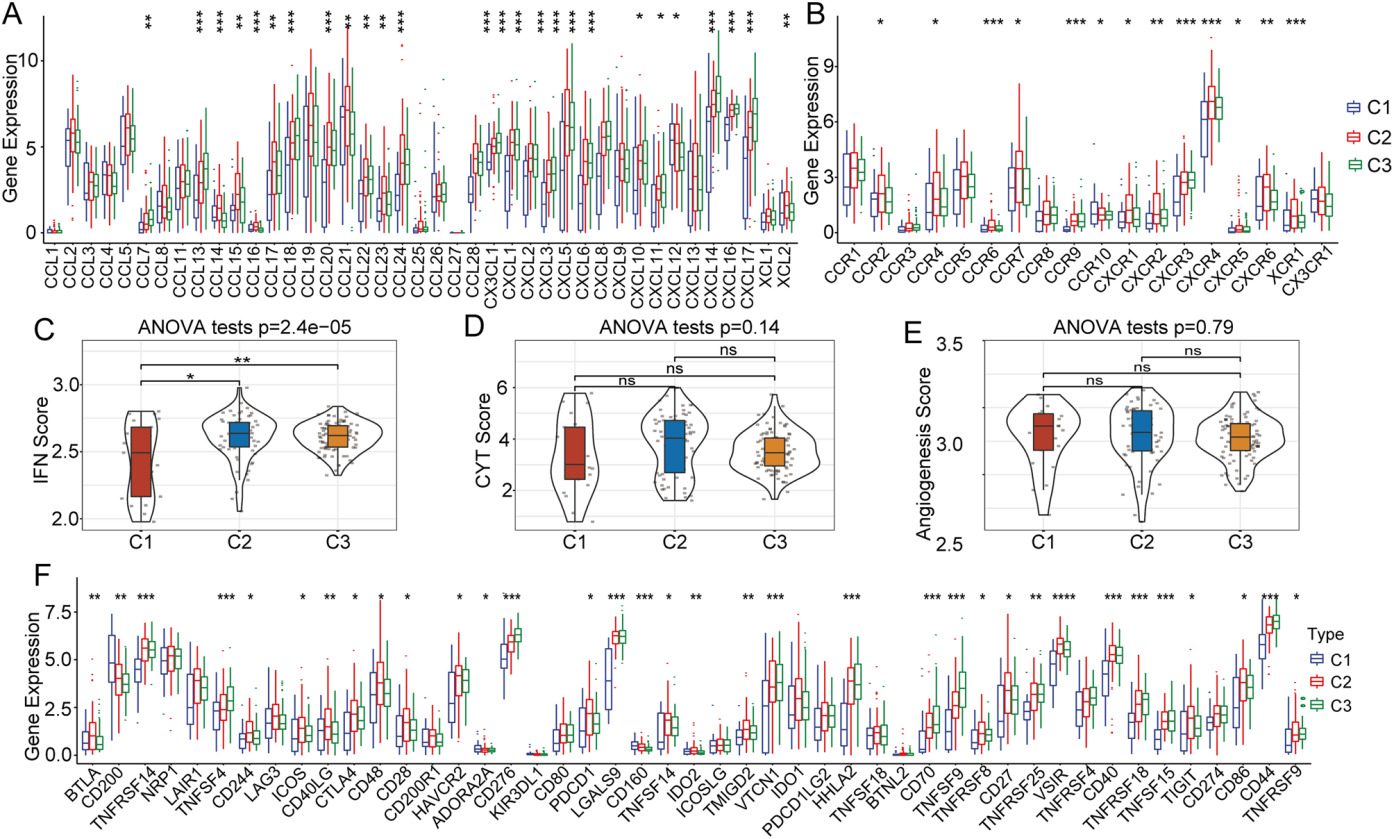
Expression of chemokines and immune checkpoint genes in different epi-PCG clusters. (**A**) Differences in the expression of chemokines among different clusters in the TCGA cohort. (**B**) Differences in the expression of chemokine receptors among different clusters in the TCGA-PACA cohort. (**C**) Differences in the distribution of IFNγ scores among different clusters in the TCGA cohort. (**D**) Differences in the cytolytic activity of T cells in different clusters. (**E**) Differences in angiogenesis scores among different clusters. (**F**) Differences in the expression and distribution of immune checkpoint genes in the TCGA-PACA cohort. A *P* value < 0.05 indicates statistical significance (*P < 0.05; **P < 0.01; ***P < 0.001).

Chemokines are best known for their roles in mediating the recruitment of immune cell subsets that affect tumor progression and immunotherapy outcomes^24^. In the TCGA-PACA cohort, comparing the expression of chemokines in the three subtypes, we found that 28 of 41 chemokines (including CCL7, CLL13, CCL14, CCL15, CCL16, CCL17, CCL18, and CCL28) had significant differences among the subtypes (Fig. 4A). In addition, we compared the expression levels of chemokine receptor genes among the different epi-PCG-related clusters. We found that 13 of the 18 chemokine receptor genes (72.22%) had significant differences in expression among the immune subtypes (Fig. 4B). These results suggested that the degree of immune cell infiltration of different subtypes may be different, and these differences may lead to differences in tumor progression and immunotherapy effects.

CD8 + T cells in the TME can produce interferon-γ (IFNγ), which can stimulate the upregulation of programmed cell death protein 1/programmed cell death ligand 1 (PD-1/PD-L1) and indoleamine 2,3-dioxygenase 1 (IDO1) gene expression^25,26^. Previous studies have shown that the upregulation of IDO1 expression is positively correlated with poor prognosis and tumor progression^27,28^. We extracted Th1/IFNγ gene signatures from a previous study and calculated the IFNγ score of each sample using the ssGSEA method^29^. We discovered significant differences in IFNγ scores among the subgroups, and C1 had the lowest scores among the three clusters (Fig. 4C). The average value of lymphocyte-derived granzyme A (GZMA) and perforin (PRF1) expression levels was used to evaluate the immune cytolytic activity (CYT)^30^. There were no significant differences among the three clusters (Fig. 4D). The angiogenesis-related gene set was obtained from a previous study, and the angiogenesis score of each patient was evaluated. We found no significant differences among the different clusters, as shown in Fig. 4E. Furthermore, we analyzed the differences in 47 immune checkpoint-related genes among the various immune subtypes^29^. We found 34 immune checkpoint genes with significant differences, including BTLA, CD200, TNFRSF14, TNFSF4CD244, ICOS, CD40LG, CTLA4, CD48, CD28CD276, CD44, and CD86 (Fig. 4F). The above molecules were also analyzed in the ICGC-PAAD and GEO-LUAD cohorts, and the results are shown in Figs. S3 and S4. All the results indicated that different clusters may have different responses to immunotherapy.

### Characteristics of immune cell infiltration in different clusters

**Figure 5:**
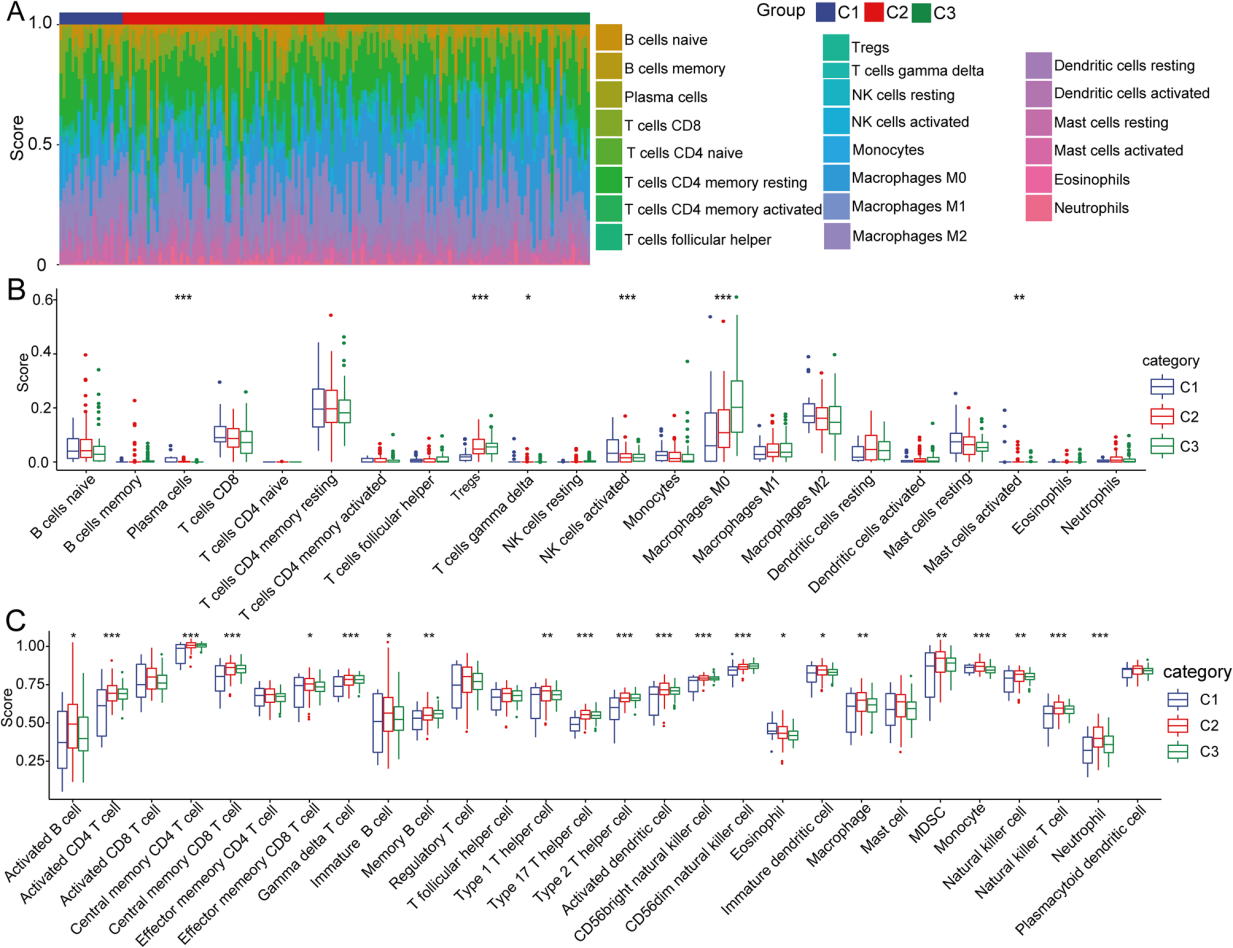
Characteristics of immune cell infiltration in different clusters. (**A**) Distribution of 22 immune cell scores in 3 subgroups. (**B**) Differences in 22 immune cell components in samples from different clusters. (**C**) Differences in ssGSEA immune scores among different clusters. (*P < 0.05; **P < 0.01; ***P < 0.001).

Tumor-infiltrating immune cells and tumor-immune interactions between the tumor and immune system have been implicated in the response to cancer immunotherapy^31^. Here, to explore the characteristics of immune cell infiltration in pancreatic cancer, we used the CIBERSORT method to assess the immune cell infiltration score. The results showed that the 3 clusters had different immune cell infiltration scores (Fig. 5A), and 6 of 22 immune cells (including plasma cells, regulatory T cells, gamma delta T cells, activated NK cells, M0 macrophages, and activated mast cells) were found to be significantly different among the different clusters (Fig. 5B). In addition, we used ssGSEA to analyze the scores of 28 immune cells previously published and then compared their differences in the 3 clusters. The results showed that 22 of the 28 immune infiltration scores had significant differences among the subtypes (Fig. 5C). Similar results were found in the GEO database (Fig. S5) and ICGC database (Fig. S6).

### Prediction of the immunotherapy effects of different epi-PCG clusters

**Figure 6:**
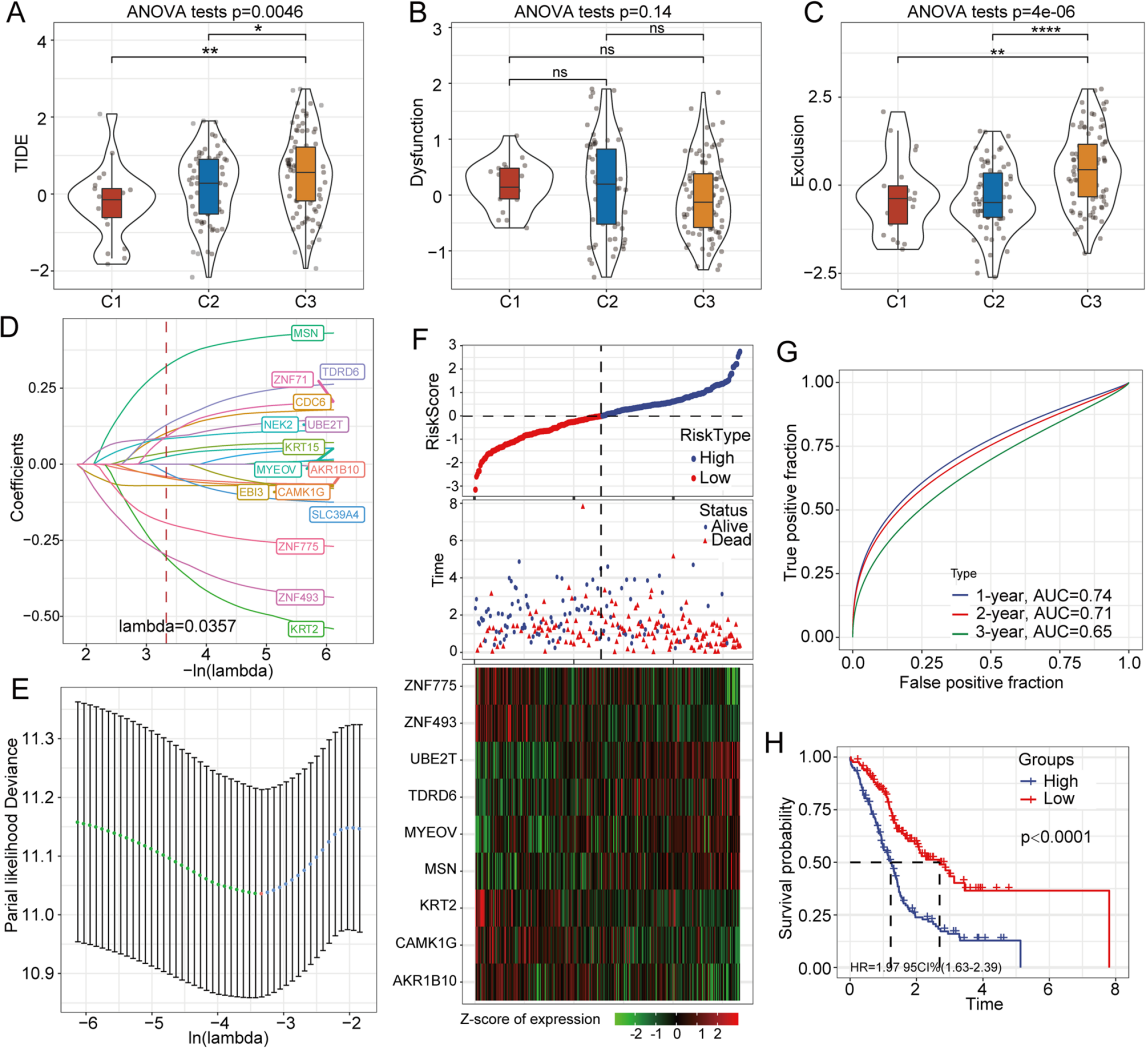
Prediction of the immunotherapy effects of different epi-PCG clusters and construction of a prognostic risk model based on epi-PCGs. (**A**) Difference in TIME scores among different epi-PCG clusters. (**B**) Difference in T cell dysfunction among the three clusters. (**C**) Difference in T cell exclusion among the three clusters. (**D**) Change trajectory of each independent variable. (**E**) Confidence interval under each lambda. (**F**) Distribution of the risk score, survival time and survival status and the expression levels of the 9 genes in the ICGC dataset. G. ROC curve and AUC of 9-gene model classification. (**H**) Survival analysis of patients with high or low risk scores in the ICGC dataset.

The low level of T cell infiltration and infiltrating T cell dysfunction in tumors are the main mechanisms of tumor immune evasion^32,33^. The generation of an accurate gene signature to assess tumor immune escape could serve as a biomarker for predicting the efficacy of immunotherapy. To explore the clinical application of the epi-PCG clusters in immunotherapy, we used TIDE software. Notably, higher tumor immune microenvironment (TIME) scores represented a greater possibility of immune escape and poor immunotherapy response. In the ICGA-PACA cohort, C1 had the lowest TIDE score compared with C2 and C3 (Fig. 6A). Although there was no difference in T cell dysfunction among the three clusters (Fig. 6B), T cell exclusion was significantly different among the three clusters (Fig. 6C). Overall, the evidence indicated that patients in C1 could benefit more from immunotherapy than those in the other two clusters.

### Construction of a prognostic risk model based on epi-PCGs

We identified 23 genes that were related to the OS of patients with pancreatic cancer. We used LASSO analysis to compress the 23 genes. First, we analyzed the change trajectory of each independent variable in the ICGC-PACA cohort, as shown in Fig. 6D. Then, we used tenfold cross-validation to build the model, and the confidence interval under each lambda is shown in Fig. 6E. When lambda = 0.0357, we identified 15 genes, which were employed for further analysis. To further reduce the number of genes, we used the stepAIC method. Finally, we reduced the 15 genes to 9 genes, and the final risk score formula was as follows: risk score = 0.401*MSN + 0.089*MYEOV + 0.23*UBE2T-0.078*AKR1B10-0.127*CAMK1G-0.549*KRT2 + 0.255*TDRD6-0.404*ZNF493-0.335*ZNF775.

### Assessment and validation of the risk model for the prognosis of patients with pancreatic cancer

To assess the predictive efficacy of the risk model for prognosis, we calculated the risk score of individual patients in the ICGC-PACA dataset. The distribution of the risk scores of the patients and the heatmap of mRNA expression are shown in Fig. 6F. Furthermore, we employed time-dependent ROC analysis to evaluate the prognostic capacity of the 9-gene model. The areas under the ROC curve (AUCs) for 1-, 2-, and 3-year OS were 0.74, 0.71 and 0.65, respectively, for the ICGC-PACA cohort (Fig. 6G). Additionally, we found that patients in the high-risk group showed poorer prognosis than patients in the low-risk group (*p* < 0.0001) (Fig. 6H). Similar procedures were performed in the GEO database (Fig. S7A–C) and TCGA database (Fig. S7D–F). All the results demonstrated that the 9-gene model had excellent performance for survival prediction. In addition, we show the grouping of patients based on risk scores in three data sets. The results show that patients can be distinguished significantly according to the risk score (Fig. S8).

### Clinical features of the high- and low-risk groups

**Figure 7:**
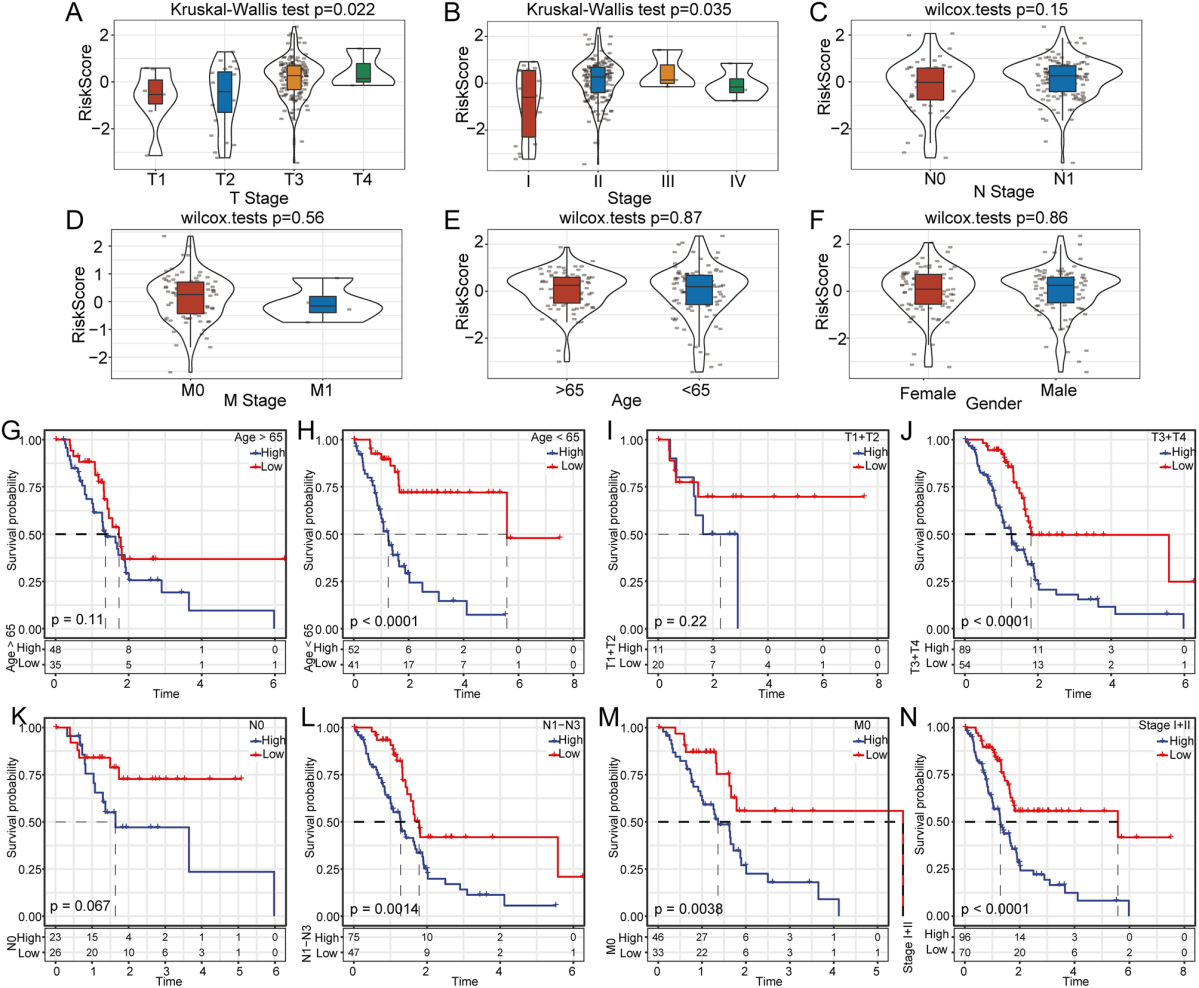
Clinical features of the high- and low-risk subgroups. (**A**–**F**) Risk score comparison of patients with different clinical features in the TCGA-PACA dataset. (**G**–**N**) Comparison of the overall survival of risk groups in subgroups with different clinical features.

After confirming the efficacy of the 9-gene model in predicting the prognosis of patients with pancreatic cancer, we further investigated whether the risk model could be used to predict OS in patients with different clinical features. In the TCGA database, we found that the risk score was significantly different in the T stage and stage subgroups (Fig. 7A,B). The risk score showed no difference in terms of N stage, M stage, age or sex (Fig. 7C–F). In addition, by comparing the OS of risk groups in subgroups with different clinical features, we found that our risk grouping also has a good prediction effect for prognosis among different clinical subgroups (Fig. 7G–N).

### The risk score is an independent risk factor for the prognosis of pancreatic cancer

**Figure 8:**
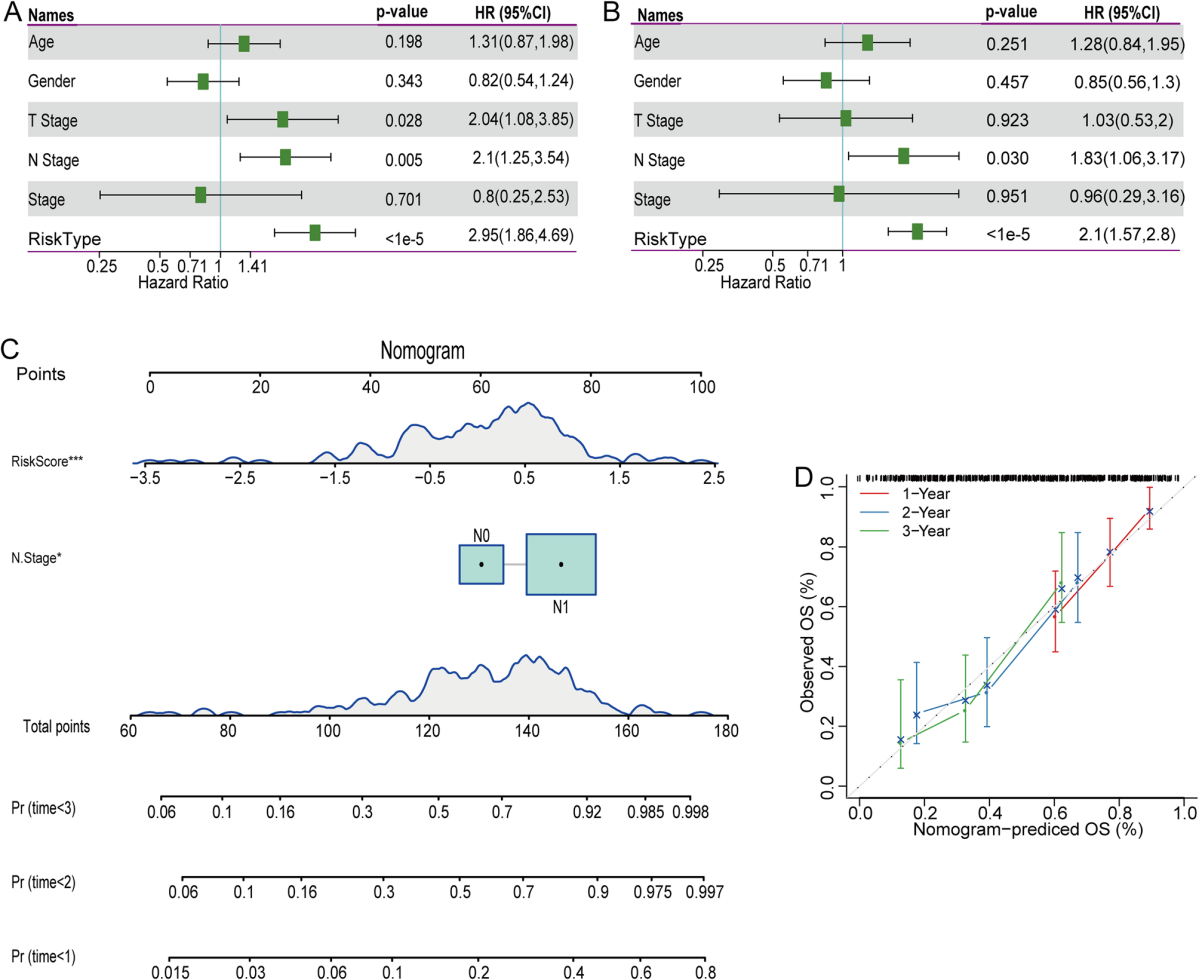
The risk score is an independent risk factor for the prognosis of pancreatic cancer. (**A**–**B**). Univariate and multivariate Cox regression analyses in the TCGA-PACA cohort. (**C**) Nomogram constructed by the risk score and clinical characteristics. (**D**) Calibration curve of the survival rate of the nomogram.

To determine the independence of the 9-gene signature model for clinical application, we performed univariate Cox regression analysis and found that the risk score was significantly related to survival. The results of multivariate COX analysis showed that risk score still played a guiding role in the prognosis and was an independent risk factor for pancreatic cancer (Fig. 8A–B). Then, we combined the clinical feature of N stage and the risk score to construct a nomogram model (Fig. 8C). According to the model results, the risk score feature has the greatest influence on survival prediction, indicating that the risk model based on 9 genes would better predict prognosis. In addition, we assessed 1, 2 and 3 years of data to visualize the performance of the nomogram (Fig. 8D). Taken together, the results suggested that the 9-gene model could serve as a classifier for prognostic stratification in clinical application.

## Discussion

Epigenetic modifications do not change the DNA sequence but alter the expression of genes. There are many mechanisms of epigenetic regulation. Histone modification, especially histone methylation, is the most interesting type of epigenetic modification in pancreatic cancer occurrence and progression^34^. KDM6A and MLL2 were found to be the most frequently altered histone methylation regulatory genes in pancreatic cancer through whole genome sequencing^35^. In this study, through transcriptome analysis of TCGA-PAAD cohort, we identified 363 epigenetic PCGs (epi PCGs). Moreover, to explore the epigenetic characteristics of PCGs induced by histone modification, we analyzed the distribution characteristics of promoters and enhancers of epi-PCGs modified by different histones in the genome. This evidence of characterization of epigenetic dysfunction would contribute to advancing our understanding of the mechanisms of pancreatic tumorigenesis.

Here, based on the epi-PCGs, we constructed three prognostic molecular clusters that had differences in OS in pancreatic cancer based on the TCGA, ICGA and GEO databases. The tumor immune microenvironment of PDAC has three important characteristics: (1) lack of immunogenicity and low immune response, indicating insufficient immune activation and excessive immunosuppression; (2) The high heterogeneity of TME in PDAC and (3) rich and dense desmosome matrix. Among them, T cells are important effectors of immune response and regulators of immunosuppression (36). In addition, there are different crosstalk between T cells and almost all components in PDAC TME. These facts make T cells become targets that cannot be ignored in immunotherapy research, including immunocheckpoint inhibitors. Our results showed that the expression of chemokines and immune checkpoint genes in different epi PCG clusters were significantly different. We also used CIBERSORT method to systematically analyze the characteristics of immune cell infiltration in different clusters. Almost all types of T cells have different degrees of infiltration in the three subtypes. All the evidences provide reference for selecting patients who are more likely to benefit from immunotherapy. The CIBERSORT method has been popularly used in triple-negative breast cancer^37^, oral cancer^38^, stomach adenocarcinoma^39^, lung adenocarcinoma^40^, head and neck cancer^41^, etc. to evaluate the immune cell composition in the TME. Importantly, a better understanding of the characteristics of the TME promotes the development of immunotherapy in patients with cancer.

Considering the individual heterogeneity of epigenetic modification, it is necessary to quantify the epigenetic modification of individual patients. Thus, we constructed a scoring system based on the epi-PCGs. The scoring tool could predict the prognosis of patients with pancreatic cancer. Here, we employed multiple omics data, including the mRNA, m6A, m5C, and m1A gene expression data of pancreatic cancer from the TCGA, GEO, and ICGC databases, histone modification information and differentially expressed PCGs between the human pancreatic cancer line PANC-1 and healthy tissues. The obvious advantage of multiple omics analysis compared with gene expression profiling is that it can better show the regulatory complexity of biological phenotypes.

In conclusion, we constructed a 9-gene prognostic risk model based on epi-PCGs that was an independent risk factor and might be used as an effective classifier to predict the prognosis and response to immunotherapy of patients with pancreatic cancer.

## Methods

### Data processing

This study is based on the analysis of histone modification data and transcriptome data of TCGA, ICGC and GEO. We downloaded the clinical information and gene expression data of pancreatic cancer samples from The Cancer Genome Atlas (TCGA), International Cancer Genome Consortium (ICGC) and Gene Expression Omnibus (GEO) databases. In total, 8 pancreatic cancer cohorts (GSE21501, GSE28735, GSE57495, GSE62452, GSE71729, GSE85916, TCGA-PACA, and ICGC-PACA) were gathered for further analysis. The clinical statistics of the processed samples are shown in the Supplementary Table 1.

Furthermore, we obtained 6 types of histone-modified replicated narrowPeak data (including H3K27ac, H3K27me3, H3K36me3, H3K4me1, H3K4me3 and H3K9me3) of the human pancreatic cancer cell line PANC-1 and normal pancreas from the Encyclopedia of DNA Elements (ENCODE) database.

### Identification of epigenetically dysregulated PCGs

To explore the role of epigenetic changes in pancreatic cancer, we used the limma R package to identify PCGs that are differentially expressed between pancreatic cancer tissues and normal control tissues. Next, we combined the GENCODE GTF file to discover the differential histone modifications. Differentially expressed PCGs between pancreatic cancer and normal samples were named PCGs. The promoters or enhancers overlapping at least one differential histone modification region were named epigenetic PCGs (epi-PCGs) or non-epi-PCGs.

### Kyoto encyclopedia of genes and genomes (KEGG) and gene ontology (GO)

KEGG and GO analyses are the most common algorithms that integrate the functional information of genes^42–44^. Here, we employed KEGG and GO analyses based on epi-PCG-related genes by the clusterProfiler package (v3.14.0) to explore the biological functions and pathways of epi-PCGs in the oncogenesis and progression of pancreatic cancer.

### Single-sample gene set enrichment analysis (ssGSEA)

ssGSEA, which is an extension of the gene set enrichment analysis (GSEA) method, is designed for individual samples that cannot be used for GSEA^45^. This procedure is similar to GSEA, but the samples are normalized and ranked by gene expression values. The enrichment score was calculated using the empirical cumulative distribution function (CDF)^45^. To assess the association between the risk scores and biological functions, we used the gene expression data to perform ssGSEA with the GSVA R package and calculated the enrichment scores of each sample on different functions.

### Cell-type identification by estimating relative subsets of RNA transcripts (CIBERSORT)

To explore the characteristics of immune subsets in the TME, we used the CIBERSORT package to quantify relative cell fractions from the gene expression data of cellular mixtures (http://cibersort.stanford.edu^46^,^47^). Samples with *p* < 0.05 in CIBERSORT analysis were used for further analysis. Student’s t test was used to compare differences in 22 types of immune cells between different epi-PCG clusters.

### Least absolute shrinkage and selection operator (LASSO)

The LASSO method was first proposed by R Tibsirani in 1997. It is used for variable selection and compression in a linear regression context^48^. In this method, the absolute values of the parameters are constrained by a constant that shrinks coefficients and makes some coefficients tend to 0. Thus, the method is more accurate than stepwise selection. In this study, the Glmnet R package was used for LASSO Cox regression analysis. First, the change track of each independent variable in the ICGC dataset was analyzed. Then, we used tenfold cross-validation for model construction.

### Tumor immune dysfunction and exclusion (TIDE)

TIDE is a computation method used to mold the features of tumor immune evasion by assessing T cell dysfunction and T cell depletion by genome-wide expression characteristics^49^. To explore the difference in immunotherapeutic response among different epi-PCG clusters, we used the TIDE (http://tide.dfci.harvard.edu) method. A higher TIDE prediction score, a higher dysfunction score and a higher exclusion of T lymphocytes indicated a higher possibility of immune escape and a lower likelihood of benefit from immunotherapy.

### Statistical analysis

All data were processed using R version 3.6.1 software and Prism 7.0 (GraphPad Software, La Jolla, CA). Student’s t test was used to compare the difference between two groups. Spearman correlation analysis was used to explore the correlation between the PCG enrichment score and m6A-, m5C-, and m1A-related genes. The prognostic value of the risk model in pancreatic cancer patients was analyzed by Kaplan–Meier curves and the log-rank test. The specificity and sensitivity of the risk score were assessed with time-dependent receiver operating characteristic (ROC) curves. All statistical results with *P* values < 0.05 were considered significant.

## Supplementary Information

Supplementary Information 1.

Supplementary Information 2.

Supplementary Information 3.

Supplementary Information 4.

Supplementary Information 5.

Supplementary Information 6.

Supplementary Information 7.

Supplementary Information 8.

Supplementary Information 9.

Supplementary Information 10.

## Data Availability

The datasets used and/or analysed during the current study available from the corresponding author on request.
