# Supplementary figures and images for: Dysfunctional epigenetic protein-coding gene-related signature is associated with the prognosis of pancreatic cancer based on histone modification and transcriptome analysis
Source: Sci Rep. 2023 Jan 4;13:146. doi: 10.1038/s41598-022-27316-2 (PMC9813002; doi:10.1038/s41598-022-27316-2)

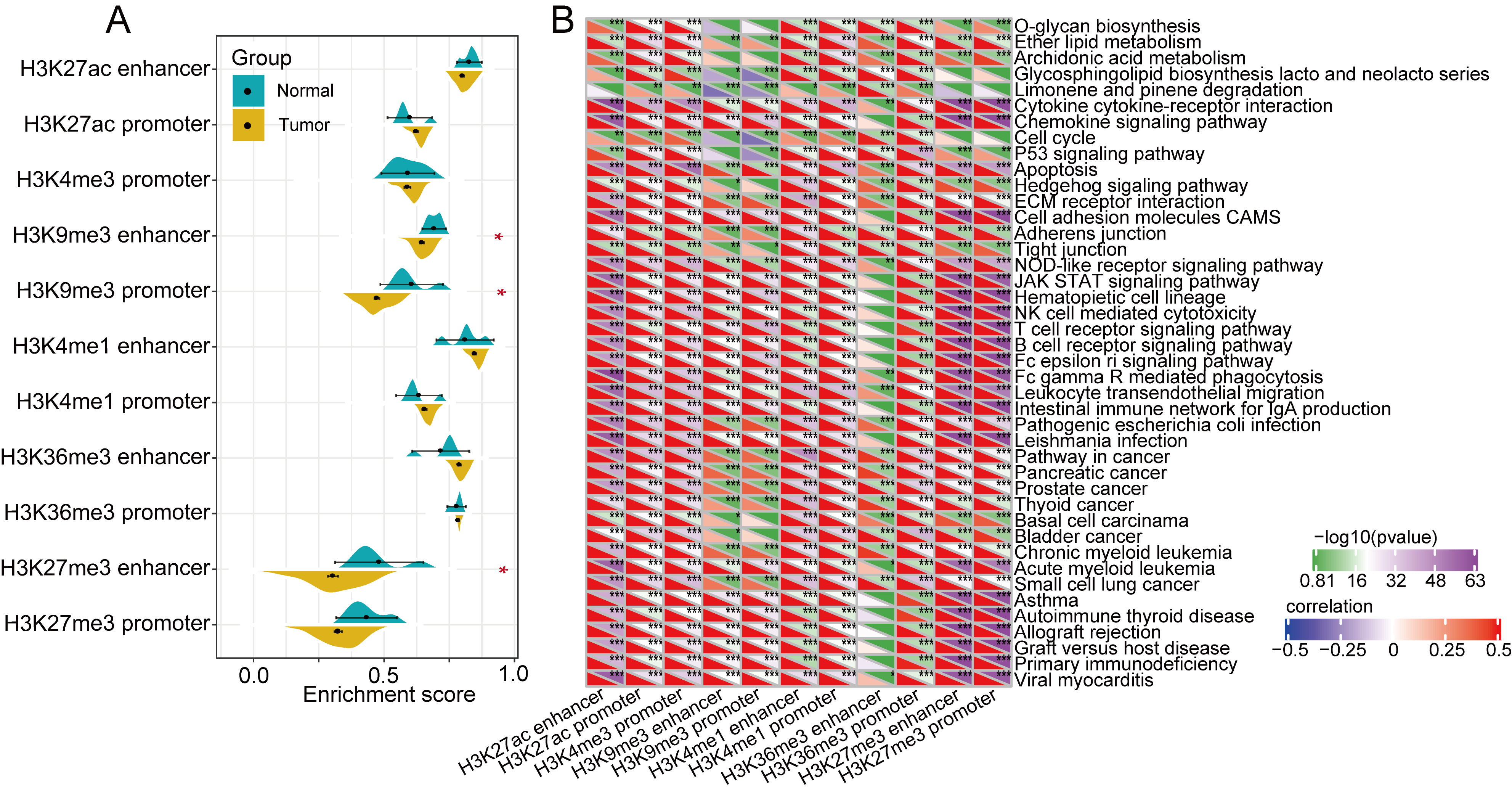

Supplement: Supplementary file 1 — Supplementary Information 1. [file 41598_2022_27316_MOESM1_ESM.tif]

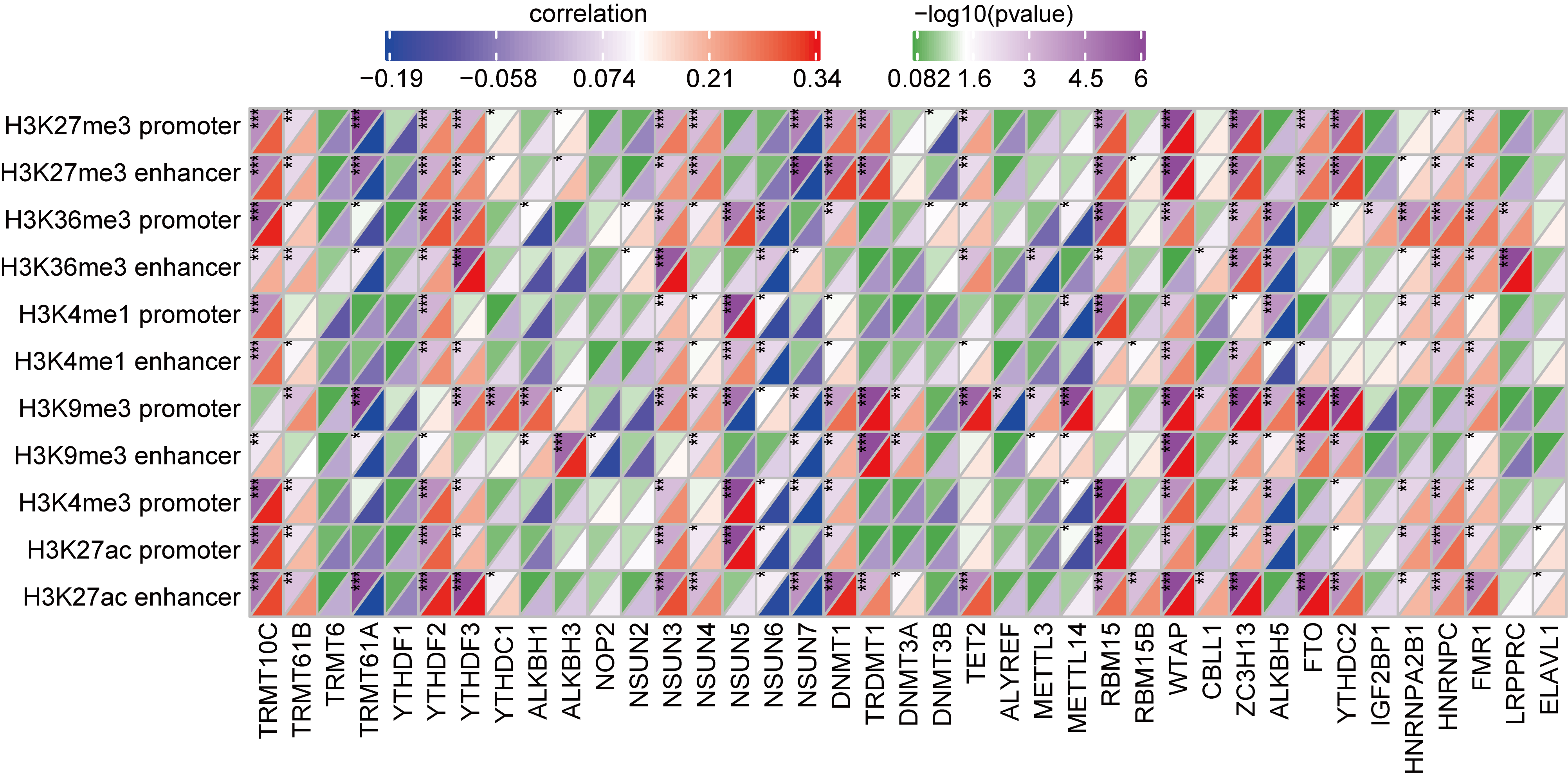

Supplement: Supplementary file 2 — Supplementary Information 2. [file 41598_2022_27316_MOESM2_ESM.tif]

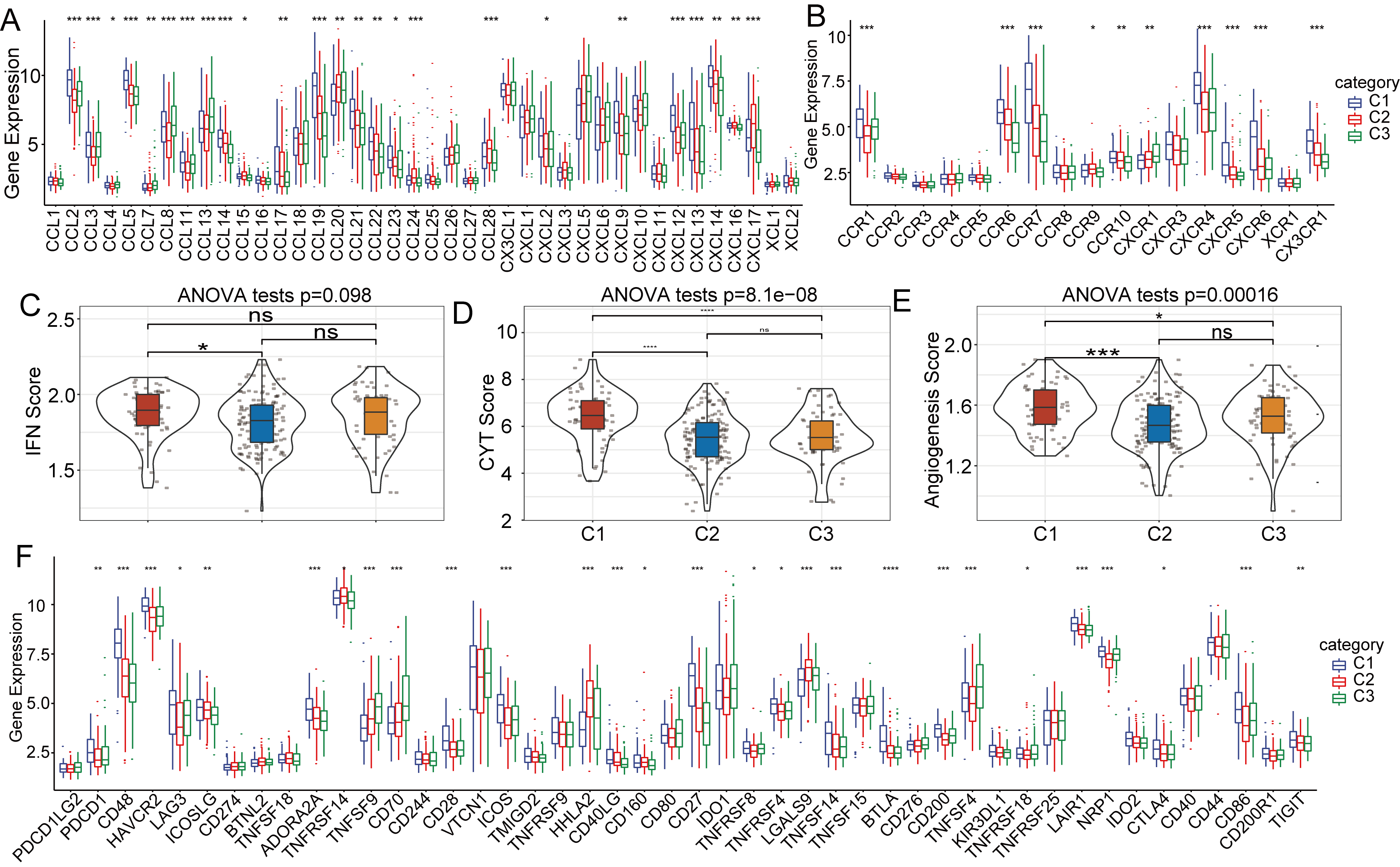

Supplement: Supplementary file 3 — Supplementary Information 3. [file 41598_2022_27316_MOESM3_ESM.tif]

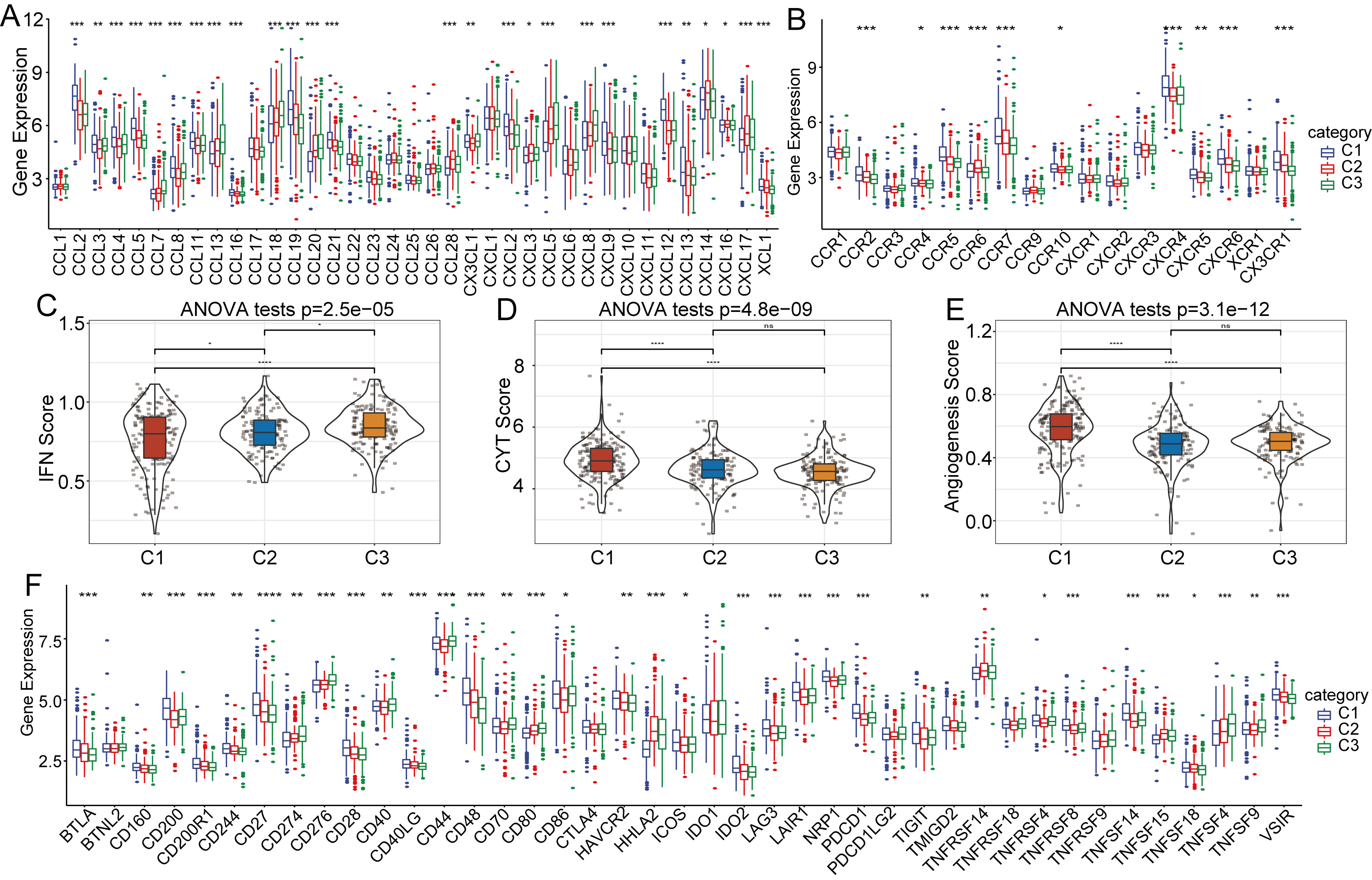

Supplement: Supplementary file 4 — Supplementary Information 4. [file 41598_2022_27316_MOESM4_ESM.tif]

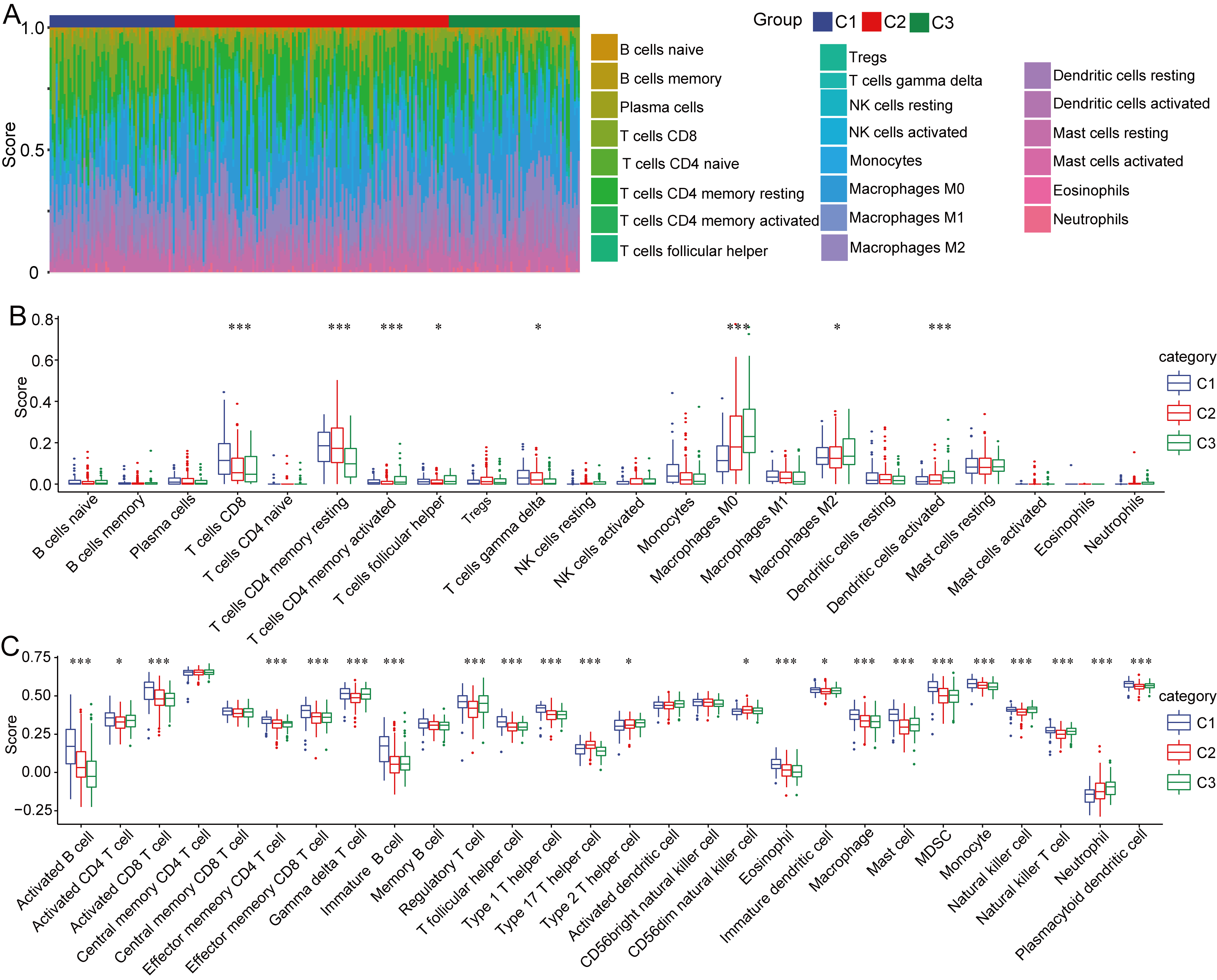

Supplement: Supplementary file 5 — Supplementary Information 5. [file 41598_2022_27316_MOESM5_ESM.tif]

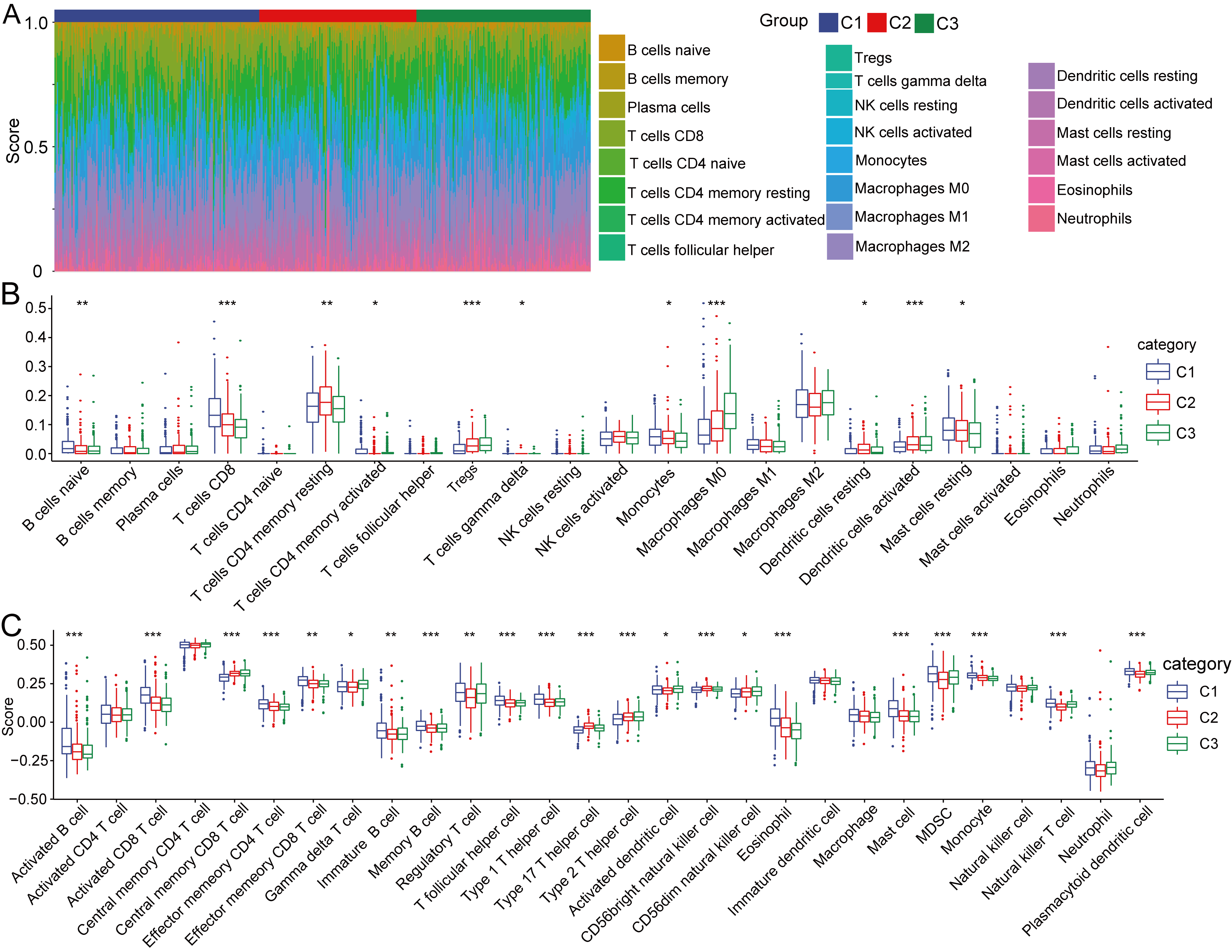

Supplement: Supplementary file 6 — Supplementary Information 6. [file 41598_2022_27316_MOESM6_ESM.tif]

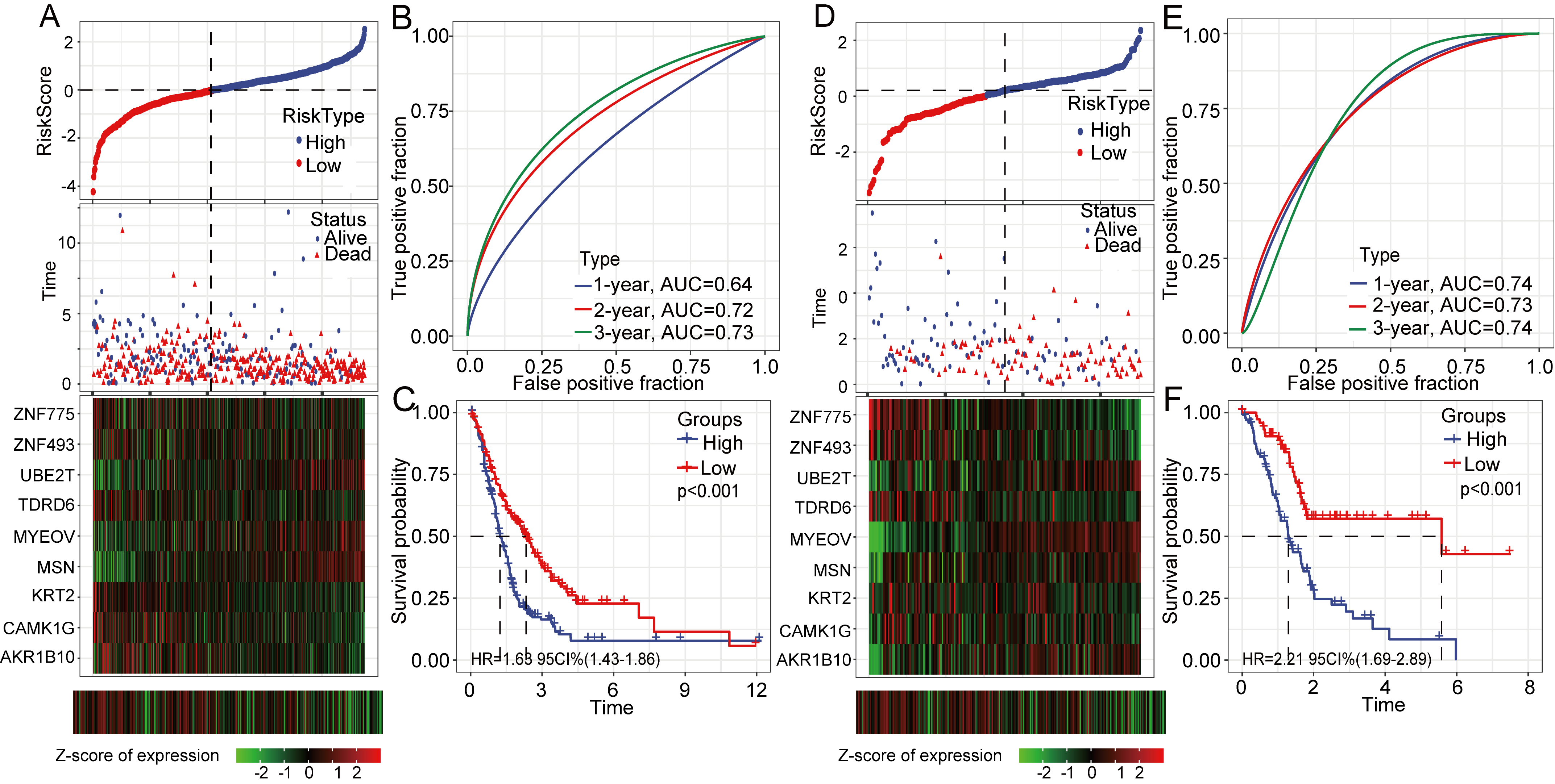

Supplement: Supplementary file 7 — Supplementary Information 7. [file 41598_2022_27316_MOESM7_ESM.tif]

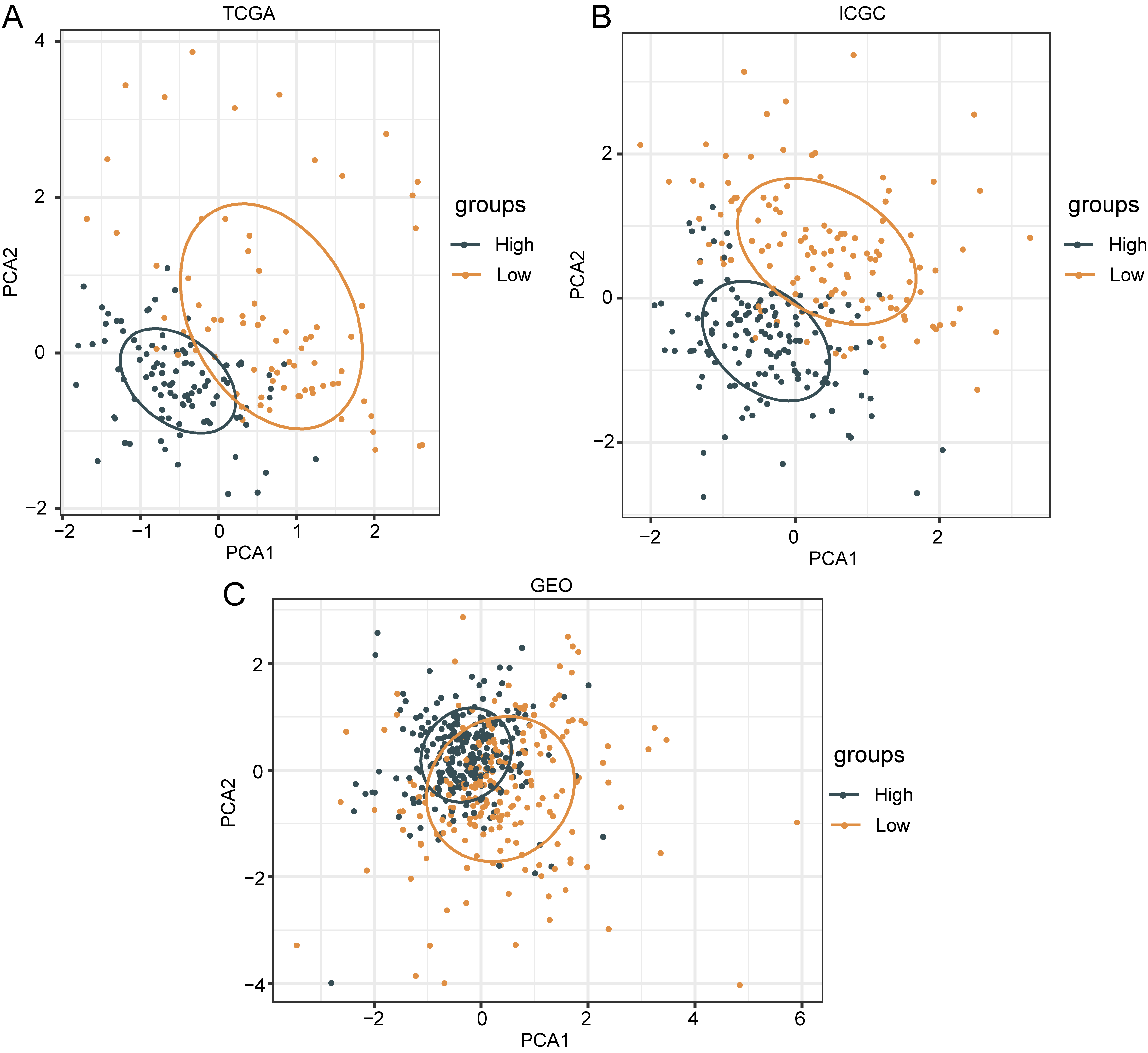

Supplement: Supplementary file 8 — Supplementary Information 8. [file 41598_2022_27316_MOESM8_ESM.tif]
