# Supplementary material for: Dysfunctional epigenetic protein-coding gene-related signature is associated with the prognosis of pancreatic cancer based on histone modification and transcriptome analysis
Source: Sci Rep. 2023 Jan 4;13:146. doi: 10.1038/s41598-022-27316-2 (PMC9813002; doi:10.1038/s41598-022-27316-2)
Supplement: Supplementary file 9 — Supplementary Information 9. [file 41598_2022_27316_MOESM9_ESM.docx]

**Supplementary figure legends**

**Figure S1.** Analysis of the enrichment of dysregulated epi-PCGs. A. ssGSEA of dysregulated epi-PCGs. B. KEGG pathway enrichment score for each sample.

**Figure S2.** Relationship between dysregulated epi-PCGs and RNA modification.

**Figure S3.** Expression of chemokines and immune checkpoint genes among different epi-PCG clusters in the ICGC dataset. A. Differences in the expression of chemokines among different clusters in the ICGC dataset. B. Differences in the expression of chemokine receptors among different clusters in the ICGC dataset. C. Differences in the distribution of IFNγ scores among different clusters in the ICGC dataset. D. Differences in the cytolytic activity of T cells in different clusters. E. Differences in angiogenesis scores among different clusters. F. Differences in the expression and distribution of immune checkpoint genes in the ICGC dataset. A P value <0.05 indicates statistical significance (*P<0.05; **P<0.01; ***P<0.001).

**Figure S4.** Expression of chemokines and immune checkpoint genes among different epi-PCG clusters in the GEO dataset. A. Differences in the expression of chemokines among different clusters in the GEO dataset. B. Differences in the expression of chemokine receptors among different clusters in the GEO dataset. C. Differences in the distribution of IFNγ scores among different clusters in the GEO dataset. D. Differences in the cytolytic activity of T cells in different clusters. E. Differences in angiogenesis scores among different clusters. F. Differences in the expression and distribution of immune checkpoint genes in the GEO dataset. A P value <0.05 indicates statistical significance (*P<0.05; **P<0.01; ***P<0.001).

**Figure S5.** Characteristics of immune cell infiltration among different clusters in the GEO dataset. A. Distribution of 22 immune cell scores in the 3 subgroups. B. Differences in 22 immune cell components in samples from different clusters. C. Differences in ssGSEA immune scores among different clusters.

**Figure S6.** Characteristics of immune cell infiltration among different clusters in the ICGC dataset. A. Distribution of 22 immune cell scores in the 3 subgroups. B. Differences in 22 immune cell components in samples from different clusters. C. Differences in ssGSEA immune scores among different clusters.

**Figure S7.** Assessment and validation of the risk model for the prognosis of patients with pancreatic cancer. A. Distribution of the risk score, survival time and survival status and the expression levels of the 9 genes in the GEO dataset. B. ROC curve and AUC of 9-gene model classification. C. Survival analysis of patients with high or low risk scores in the GEO dataset. D. Distribution of the risk score, survival time and survival status and the expression levels of the 9 genes in the TCGA dataset. E. ROC curve and AUC of 9-gene model classification. F. Survival analysis of patients with high or low risk scores in the TCGA dataset.

Figure S8. Functional enrichment analysis of the gene set. A. Clustering of the correlation coefficients between the KEGG pathways with a risk score correlation greater than 0.4 and the risk score. B. The association between the KEGG pathways and a risk score correlation greater than 0.4 in the ssGSEA score changes in each sample as the risk score increases.

**Figure S8.** PCA analysis based on risk score in three data sets.

**Supplementary Table legends**

Supplementary Table 1: Clinical information statistics of the cohort analyzed in this study.
